# Kinetic patterns of benign and malignant breast lesions on contrast enhanced digital mammogram

**DOI:** 10.1371/journal.pone.0239271

**Published:** 2020-09-17

**Authors:** Jer-Shyung Huang, Huay-Ben Pan, Tsung-Lung Yang, Bao-Hui Hung, Chia-Ling Chiang, Meng-Yuan Tsai, Chen-Pin Chou

**Affiliations:** 1 Department of Radiology, Kaohsiung Veterans General Hospital, Kaohsiung, Taiwan, ROC; 2 National Yang-Ming University, School of Medicine, Taipei, Taiwan, ROC; 3 Department of Radiology, Golden Hospital, Pingtung, Taiwan, ROC; 4 Department of Medical Laboratory Sciences and Biotechnology, Fooyin University, Kaohsiung, Taiwan, ROC; Mayo Clinic College of Medicine, UNITED STATES

## Abstract

**Purpose:**

To evaluate the kinetic patterns of benign and malignant breast lesions using contrast-enhanced digital mammogram (CEDM).

**Methods:**

Women with suspicious breast lesions on mammography or ultrasound were enrolled. Single-view mediolateral oblique (MLO) CEDM of an affected breast was acquired at 2, 3, 4, 7, and 10 min after injection of contrast agent. Three readers visually and semi-quantitatively analyzed the enhancement of suspicious lesions. The kinetic pattern of each lesion was classified as persistent, plateau, or washout over two time intervals, 2–4 min and 2–10 min, by comparing the signal intensity at the first time interval with that at the second.

**Results:**

There were 73 malignant and 75 benign lesions in 148 patients (mean age: 52 years). Benign and malignant breast lesions showed the highest signal intensity at 3 min and 2 min, respectively. Average areas under receiver operating characteristic (ROC) curve for diagnostic accuracy based on lesion enhancement at different time points were 0.73 at 2 min, 0.72 at 3 min, 0.69 at 4 min, 0.67 at 7 min, and 0.64 at 10 min. Diagnostic performance was significantly better at 2, 3, and 4 min than at 7 and 10 min (all p < 0.05). A washout kinetic pattern was significantly associated with malignant lesions at 2–4 min and 2–10 min frames according to two of the three readers’ interpretations (all p ≤ 0.001).

**Conclusion:**

Applications of optimal time intervals and kinetic patterns show promise in differentiation of benign and malignant breast lesions on CEDM.

## Introduction

Although digital mammography (DM) and digital breast tomosynthesis (DBT) are effective tools for both screening and diagnosis of breast cancers, these anatomic modalities cannot detect all forms of breast cancers [1, 2]. Malignancies usually undergo a highly angiogenic process, and contrast agent is preferentially taken up by cancerous tissue rather than normal, thus explaining the value of supplemental functional modalities like breast dynamic contrast-enhanced MRI (DCE-MRI) and contrast-enhanced digital mammogram (CEDM). DCE-MRI can detect additional breast cancers even after DM and breast clinical examination in average-risk and high-risk women [3–5]. CEDM is superior to DM and DBT in accuracy, and comparable to DCE-MRI in assessing breast malignancy [5].

Breast lesion enhancement on MRI can also be characterized qualitatively by assessing early enhancement and subsequent trend of the dynamic kinetics curve [6, 7]. Early enhancement phase for morphological characteristics on MRI enables the most predictive feature of interpretation and kinetics patterns using late phase MRI may assist in determining malignancy [6, 7]. Persistent late enhancement on breast MRI is generally considered benign, while washout is suggestive of malignancy [6, 7]. The biggest difference between CEDM and DCE-MRI is that DCE-MRI has a unique opportunity for comparing images at several different time points [5, 8]. Thus, determination optimal delay time window for target lesion is critical for examination protocol of CEDM. Moreover, kinetic change of tissue perfusion may explain the difference in signal intensity enhancement of breast cancer on CEDM between 2 min mediolateral oblique (MLO) view and 4 min craniocaudal (CC) view in the previous CEDM study [9]. The primary goal of the analysis was to determine the optimal time interval of breast cancer using DCE-mammogram. A secondary aim was to understand the kinetic patterns of breast cancer on CEDM.

## Materials and methods

This prospective study was the Health Insurance Portability and Accountability Act (HIPAA) compliant and approved by the Kaohsiung Veterans General Hospital institutional review board (VGHKS11-CT4-16). Written informed consent was obtained from all participants.

### Subject inclusion

Women with suspicious breast lesions (BI-RADS 4 or 5) on mammography or breast ultrasound between July 2014 and October 2016 were enrolled before biopsy or surgery in this multi-reader multi-case (MRMC) study. Women with breast implants or who refused biopsy were excluded. All participants underwent percutaneous breast core needle biopsy or excision surgery for suspicious lesions. Histopathology results of suspicious lesion were correlated with all imaging findings.

### DCE-mammogram technique

A single-view MLO DCE-mammogram along with DBT of an affected breast was obtained using a prototype unit modified from commercially available mammography (Selenia Dimensions®, Hologic, Bedford, MA). The unit uses a tungsten tube with aluminum and copper filtration. An amorphous selenium detector 24 cm x 29 cm was used. To obtain a similar compression and position of breast imaging on MLO view within 10 minutes, we did not release the paddle, and CC view of the ipsilateral breast was not obtained.

A dual-energy DCE-mammogram was acquired with kVp and filter switching. Parameters for each image were automatically selected according to the thickness of breast tissue. Low-energy imaging was performed with either rhodium or silver filtration, using a kVp between 26 and 32; while high-energy imaging was performed with copper filtration and 49 kVp.

**Fig 1:**
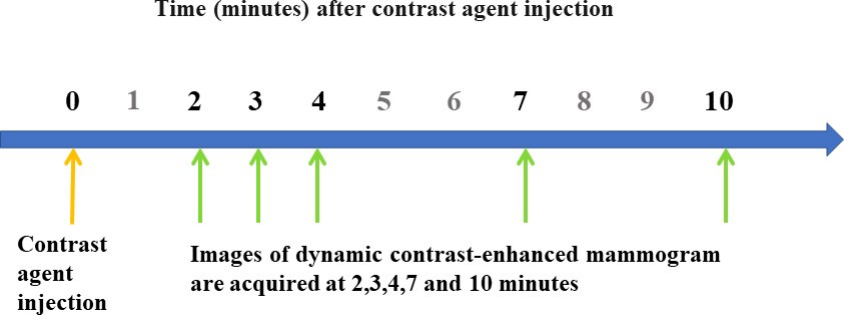
Flow chart of imaging data acquisition for dynamic contrast-enhanced digital mammogram.

A dual-energy DCE-mammogram of an affected breast was obtained after intravenous injection of 1.5 mL/kg of body weight of iohexol (Omnipaque 300, GE Healthcare, 300 mg I/mL) through the antecubital vein. The injection rate was 3 mL/sec using an automated power injector pump. The breast was uncompressed during intravenous injection. After breast compression, MLO views were acquired at 2, 3, 4, 7, and 10 min after contrast agent injection (Fig 1). All DCE-mammogram images along with DBT from 2 min to 10 min were obtained under only one single MLO compression. The effective radiation dose from a DCE-mammogram exam of a single breast was around 2.16 mSv.

### Image interpretation

**Fig 2:**
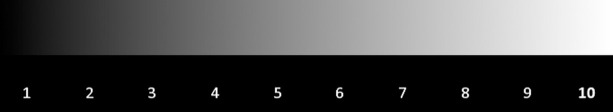
Grayscale bars with score finder above are an assumed grayscale representation of enhancement of suspicious lesion using a 10-point grayscale reference of iodine concentration within the breast lesion where pure white (score = 10) represents the highest level of contrast enhancement and black (score = 1) means poor contrast enhancement.

DM and DCE-mammograms were interpreted using a three-monitor Hologic diagnostic workstation (SecurViewDx, Hologic MA). Dual high-resolution (5MP) diagnostic grayscale medical displays were used for DM and DCE-mammogram to allow side-by-side comparison between dynamic images at different time intervals. Three board-certified breast radiologists with 30, 24, and 22 years of post-residency mammogram reading experiences, and four years of experience in CEDM served as readers (readers A, B, and C, respectively). Every reader reviewed DCE-mammograms 2–10 min independently. The non-contrast DM was also available when reading the DCE-mammogram. All readers visually and semi-quantitatively analyzed the enhancement of suspicious lesions using a 10-point Grayscale reference bar, transformed from 8-bit gray-level range using ImageJ imaging software (ver. 1.43; National Institutes of Health, Bethesda, MD, USA) to determine lesion enhancement in all DCE-mammograms (Fig 2). A research assistant recorded the readers’ signal-intensity scores at five time intervals and made sure that the same target lesions were interpreted by readers. When scoring grey values of lesions, only integers were allowed. All readers had the same viewing circumstances including window level settings, room lighting, and monitor equipment.

### Data analysis

The kinetic time-intensity patterns between early enhancement (2 min) and two delayed post-contrast imaging sessions (4 min and 10 min) were further classified into three categories: type 1, persistent (increased score), type 2, plateau (same score), and type 3, washout (decreased score). Two kinetic patterns at 2–4 min and 2–10 min were categorized for each lesion. Interindividual correlation of signal-intensity score and kinetic pattern in each target lesion was analyzed by using intraclass correlation coefficients (ICCs) with two-way mixed-effects. We considered ICC values of reproducibility less than 0.4 as poor, 0.4 to 0.59 fair, 0.6 to 0.75 good, and greater than 0.75 excellent.

In this multi-reader multi-case (MRMC) study, the receiver operating characteristics (ROC) and the confidence intervals (CIs) of individual reader and across all readers were computed using the iMRMC software with a large sample approximation and pooled average (version 4.0.3, Division of Imaging, Diagnostics, and Software Reliability, OSEL/CDRH/FDA, Silver Spring, MD) [10]. The area under ROC curve (AUC) method was employed to analyze the prediction performance of contrast enhancement. The optimal cut-off signal-intensity values for maximizing specificity and sensitivity were determined using the Youden index (MedCalc Statistical Software version 13.1, Osten, Belgium). The significance of kinetic patterns and final pathology was examined using a chi-square test for trend, modelling the kinetic pattern categories as a one degree-of-freedom linear term. Statistical tests were performed using SPSS 22 (IBM, Armonk, NY, USA). A two-sided *P*-value < 0.05 was considered statistically significant.

## Results

**Fig 3:**
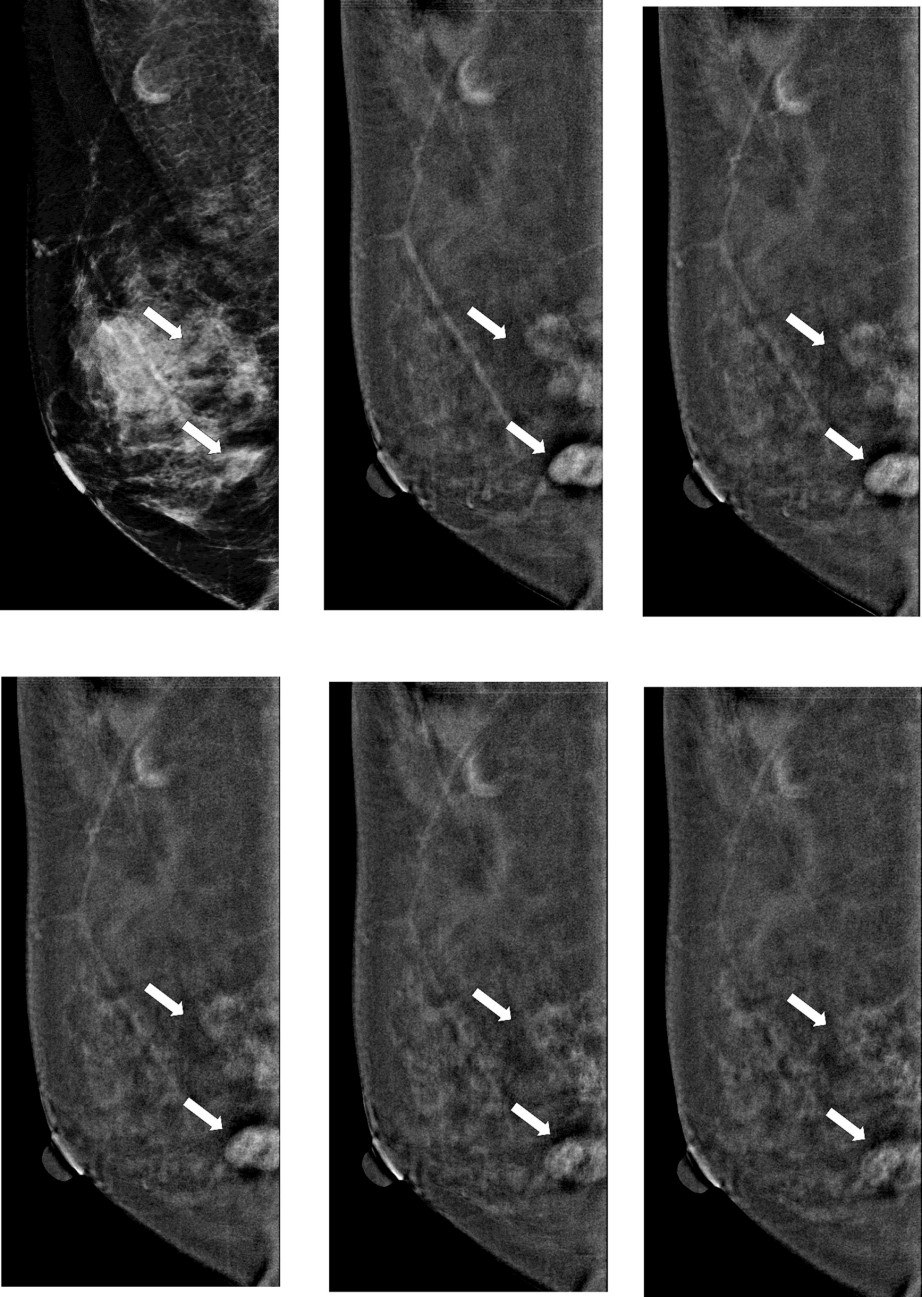
Representative washout images of malignant breast lesion on DCE-mammogram of a 52-year-old woman with invasive breast cancer in right breast. (a) Digital mammogram in MLO view before contrast agent injection shows focal asymmetry (arrows); (b-f) subtracted images at 2, 3, 4, 7 and 10 min after contrast agent injection. Subtracted images of breast malignancies (arrows) show early marked contrast enhancement and late-phase washout pattern.

**Fig 4:**
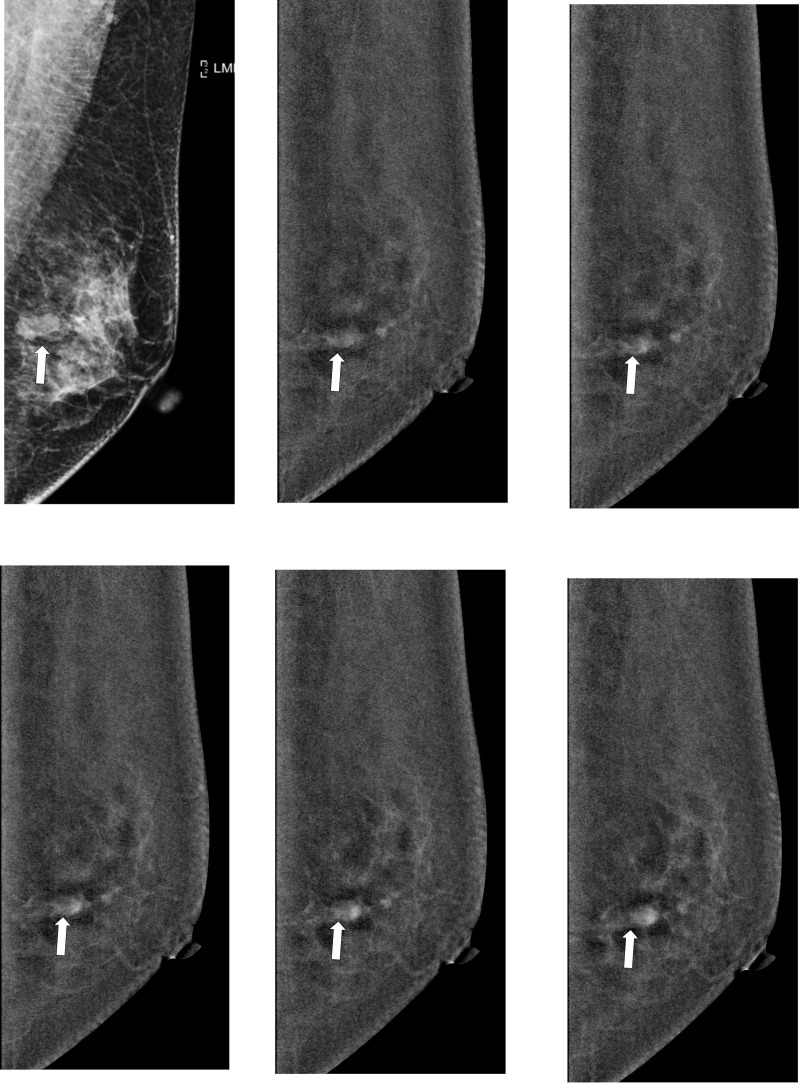
Representative persistent images of benign breast lesion on DCE-mammogram of a 45-year-old woman with fibroadenoma in left breast. (a) Digital mammogram in MLO view before contrast agent injection shows a mass (arrow); (b-f) subtracted images at 2, 3, 4, 7 and 10 min after contrast agent injection. Subtracted images of fibroadenoma (arrow) show early moderate contrast enhancement and late-phase persistent pattern.

During the study period, 148 patients met inclusion and exclusion criteria (Table 1). A total of 32 patients were excluded because of incomplete examination or pathology data. The mean age of participants was 52 years (range: 31–62). About 72% of participants were initially classified as BI-RADS 4 or 5 according to DM findings, other 28% were enrolled due to suspicious breast lesions on ultrasound. About 76% of women did not have breast symptoms, and lesions were incidentally found on screening mammogram or ultrasound. The most common imaging finding on DM was mass (44%). The mean sizes of malignant and benign masses on DCE-mammogram were 1.7 cm (range: 0.5–5.6 cm) and 1.3 cm (range: 0.8–2.6 cm), respectively. There were 73 breast cancers (53 invasive and 20 non-invasive) and 75 benign breast lesions (Figs 3 and 4, respectively). The mean pathology size of invasive cancers found at surgery was 1.5 cm (range: 0.1 to 5.6 cm).

**Table 1:** Characteristics of 148 breast lesions.

Mammography BI-RADS category	N	%
1	28	19
2	0	0
3	14	9
4A	55	37
4B	25	17
4C	10	7
5	16	11
**Symptoms**		
Benign symptomatic lesions	9	6
Malignant symptomatic lesions	26	18
Asymptomatic lesions	113	76
**Breast density present on mammogram**		
a. Fatty	0	0
b. Scattered fibroglandular	11	7
c. Heterogeneously dense	120	82
d. Extremely dense	17	11
**Main breast lesion findings**		
Microcalcification	27	18
Mass	65	44
Architectural distortion	29	20
Focal asymmetry	27	18
**Histological diagnoses**		
Invasive breast cancer	53	36
Non-invasive breast cancer	20	13
Benign breast lesion	75	51

**Fig 5:**
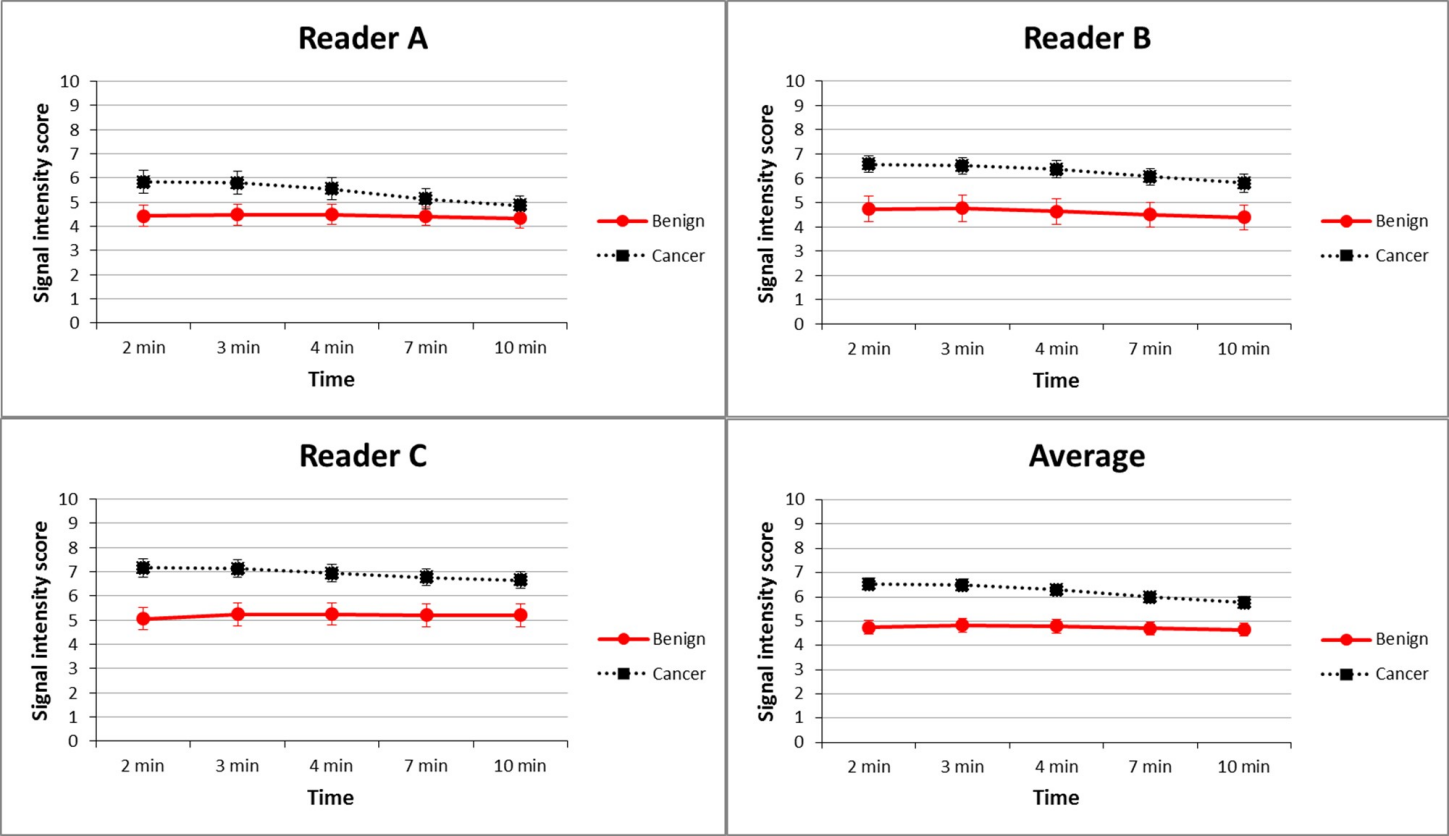
DCE-mammogram demonstrating mean signal-intensity score of benign and malignant breast lesions from three readers and their average. Malignant lesions show a significantly higher score than benign lesions on DCE-mammogram at all five time intervals (all p < 0.05) in three readers and their average. Figures present mean signal-intensity score and error bars present 95% CI. CI, confidence intervals.

Signal-intensity scores of breast lesions at five different time intervals among the three readers are shown in Fig 5. The highest average mean scores of malignant (6.52 ± 1.81) and benign lesions (4.82± 1.81) across three readers were at 2 min and 3 min, respectively. The signal-intensity scores of malignant lesions were significantly higher than those of benign lesions at all five time intervals in three readers and the average mean score (all p < 0.001). Inter-reader reliability for scoring the signal intensity of lesions at different time intervals was analyzed. Results of signal-intensity scores at different time intervals showed good to excellent (ICC range = 0.69–0.90) between each pair of readers; and good (ICC range = 0.62–0.75) among three readers. The highest agreement of lesion enhancement among three readers was observed at 2 min (ICC = 0.90; 95% CI: 0.84, 0.93), while the lowest agreement was found at 10 min (ICC = 0.83; 95% CI: 0.69, 0.89).

**Fig 6:**
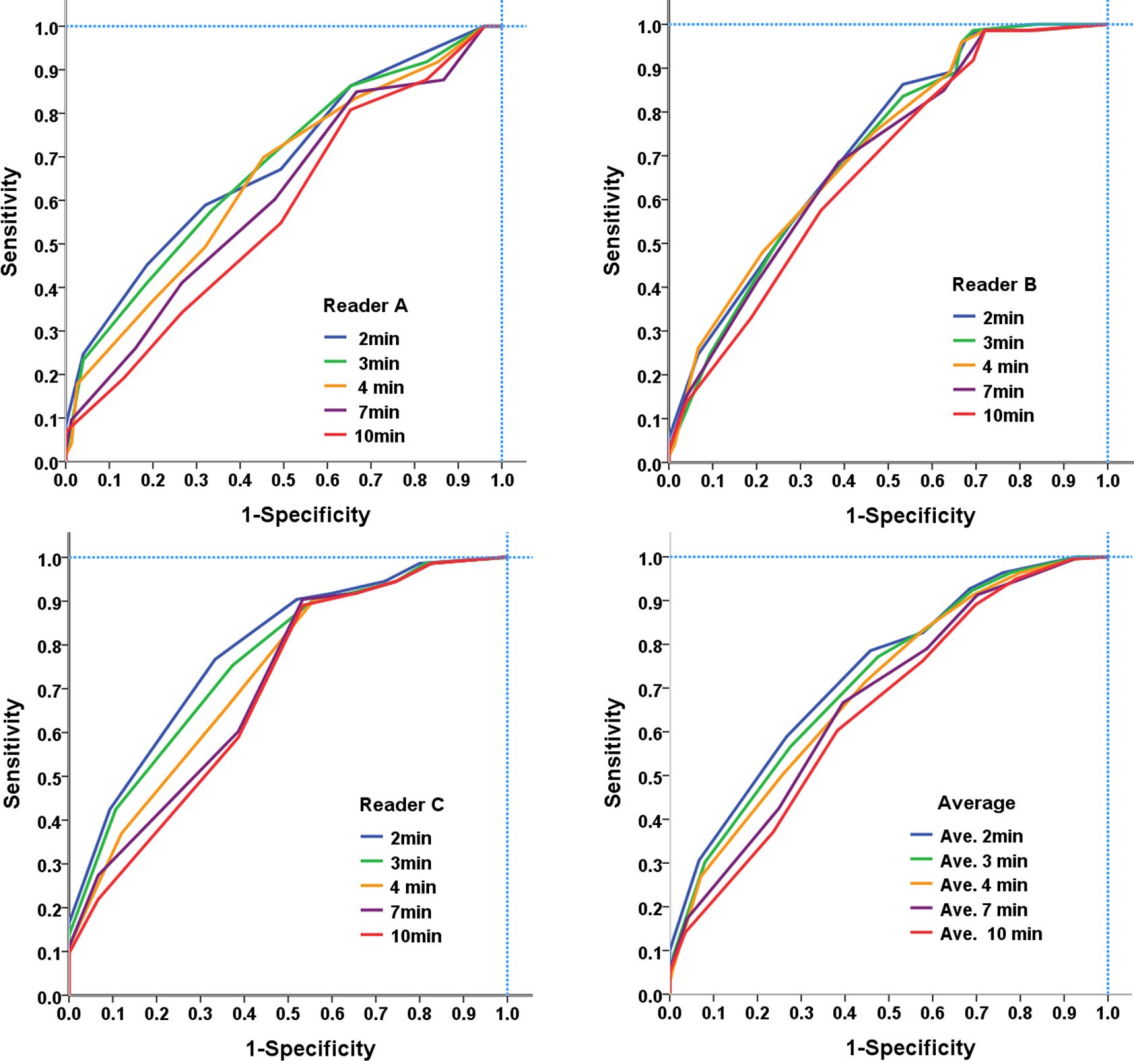
Areas under receiver operating characteristic curves (AUCs) for diagnostic performance of visual signal intensity (10-point scores) at five different time points determined by individual three readers and their average.

AUCs for distinguishing benign and malignant lesions were analyzed using the signal intensity of contrast enhancement at five different time intervals after contrast agent injection (Fig 6). Average AUCs were 0.73 at 2 min, 0.72 at 3 min, 0.69 at 4 min, 0.67 at 7 min, and 0.64 at 10 min (Table 2). Diagnostic performance was better at 2, 3, and 4 min than at 7 and 10 min (all p < 0.05), with the best at 2 min; while the reader-averaged AUC values at 2, 3, and 4 min showed no significant difference (all p > 0.05). Table 3 shows the optimal cut-off score determined for diagnostic performance (sensitivity and specificity) of lesion discrimination. The ROC analysis of three readers revealed that the optimal cut-off scores at 2, 4, and 10 min for discriminating benign and malignant lesions were 5–6, 3–5, and 2–5, respectively.

**Table 2:** Multi-Reader Multi-Case (MRMC) performance analysis: Areas under receiver operating characteristic curves (AUCs) for enhancement score of each lesion by individual readers and average of three readers.

Reader	2 min	3 min	4 min	7 min	10 min
A	0.68 (0.60–0.76)	0.68 (0.60–0.75)	0.65 (0.57–0.73)	0.61 (0.52–0.69)	0.57 (0.49–0.65)
B	0.72 (0.64–0.79)	0.71 (0.63–0.78)	0.72 (0.64–0.79)	0.69 (0.62–0.77)	0.67 (0.59–0.75)
C	0.78 (0.71–0.85)	0.76 (0.68–0.83)	0.73 (0.65–0.79)	0.71 (0.62–0.78)	0.69 (0.61–0.76)
ABC	0.73 (0.64–0.82)	0.72 (0.63–0.80)	0.69 (0.61–0.78)	0.67 (0.57–0.76)	0.64 (0.54–0.77)

Data are AUCs, and data in parentheses are 95% confidence interval. A: reader A; B: reader B; C: reader C; and ABC: average of three readers.

**Table 3:** Sensitivity, specificity of best cut-off score for contrast enhancement to differentiate between benign and malignant breast lesions on DCE-mammogram at five different time intervals.

Reader	A	B	C
2 min	>5 (59%, 68%)	>5 (86%, 47%)	>6 (77%, 67%)
3 min	>5 (58% 67%)	>5 (84%, 47%)	>6 (75%, 63%)
4 min	>4 (70% 55%)	>3 (96%, 33%)	>5(90%, 44%)
7 min	>3 (85% 33%)	>5 (69% 61%)	>5(90%, 47%)
10 min	>3 (81% 35%)	>2 (99%, 28%)	>5(89%, 47%)

Data are best cut-off score of contrast enhancement to differentiate between benign and malignancy, Numbers in parentheses are sensitivity and specificity. A: reader A; B: reader B; C: reader C.

Kinetic patterns of 148 lesions from 2–4 and 10 min were analyzed using the data of three readers. Kinetic patterns between 2–4 min also revealed poor to fair (ICC range = 0.22–0.45) inter-reader reliability between pairs of readers and fair among three readers (ICC = 0.46; 95% CI: 0.30,0.59). In contrast, kinetic patterns between 2–10 min showed higher inter-reader reliability (ICC range = 0.57–0.66) between pairs of readers and among three readers (ICC = 0.71; 95% CI: 0.62, 0.78).

The trend of kinetic patterns was significantly correlated with malignant lesions in both 2–4 min and 2–10 min time frames (both p value for trend ≤0.001) for readers A and C (Tables 4 and S1). Reader B had a marginally significant p value (0.03) for trend in 2–10 min time frame (S2 Table). At 2–4 min, the most common kinetic pattern of breast cancer was plateau among three readers (56–71%, average 64%). At 2–10 min, the most common kinetic pattern of breast cancer was washout among three readers (51–60%, average 56%). However, most benign breast lesions showed a plateau pattern among three readers in both 2–4 min (70–80%, average 74%) and 2–10 min (52–67%, average 59%) time frames. Kinetic characteristics from reader C are shown as an example. In 2–4 min time frame, the most common kinetic pattern of all lesions was plateau enhancement, presenting in 71% of breast cancer and 80% of benign lesions. Only 4% breast cancers (two DCIS and one IDC) showed a persistent increase in signal intensity at 2–4 min. In 2–10 min time frame, 51% breast cancers presented with washout pattern while 67% benign lesions still showed plateau enhancement in reader C.

**Table 4:** Kinetic patterns of DCE-mammogram between benign and malignant breast lesions using 2–4 and 2–10 min time intervals (reader C).

Kinetic patterns	Benign Lesions (N = 75)	Malignant Lesions (N = 73)	Chi-square for trend
2–4 min			p< 0.001
Persistent	11 (15%)	3 (4%)	
Plateau	60 (80%)	52 (71%)	
Washout	4 (5%)	18 (25%)	
2–10 min			p< 0.001
Persistent	14 (18%)	6 (8%)	
Plateau	50 (67%)	30 (41%)	
Washout	11 (15%)	37 (51%)	

Data are number of lesions, numbers in parentheses are percentage.

## Discussion

In the present study, we found 2 min to be the best to visualize the contrast enhancement of breast cancers and differentiate the enhancement of benign and malignant breast lesions. The time window between 2–4 min has better diagnostic performance than 7–10 min. Kinetic patterns of washout are significantly correlated with breast malignancy, especially at 2–10 min time frame. CEDM has inherent technological limitations and ethical issues in acquiring bilateral breasts images simultaneously at multi-intervals. Only a few studies about DCE-mammography have been attempted so far. This study provided the dedicated kinetic investigation and possible clinical application in the conventional bilateral CEDM exams.

Since the introduction of dual-energy CEDM, enhancement of breast lesions by an intravenous contrast agent could be imaged using a pair of high- and low-energy images on digital mammography. While early techniques utilized temporal subtraction for detecting contrast agent [11], dual-energy subtraction acquired imaging at full compression without inhibition of contrast agent uptake by breast lesions. The dual-energy technique also allows imaging in standard bilateral CC and MLO views within 10 min. CEDM protocols can vary in acquisition time, injection rate, and MLO/CC positioning protocol. Unlike MRI, CEDM did not provide an optimal time window and kinetic pattern of suspicious lesions. The European Society of Breast Imaging recommends at least two sampling intervals to evaluate the early-phase peak enhancement and kinetic patterns in breast MRI [7]. The specificity of breast MRI could be improved when kinetic patterns are applied in the interpretation [12]. Although the sensitivity of CEDM is high in breast cancer diagnosis, the specificity is less discussed.

The intensity of lesion enhancement on MRI at post-contrast 2 min is considered the most critical [7, 13]. Our study found that 2-min is the most optimal time interval in differentiating benign and malignant breast lesions. For agreement among three readers, 2-min interval can reach the best inter-readers agreement for lesion enhancement. Benign and malignant CEDM lesions showed the highest contrast enhancement at 3 and 2 min, respectively. Hence, to observe the peak contrast enhancement of breast cancer, CEDM images in MLO or CC views should be obtained at 2 min.

The kinetic patterns of DCE-MRI can be utilized to differentiate benign and malignant breast lesions [7, 13]. A washout enhancement pattern suggests malignant lesions [12, 13]. The washout of contrast enhancement could also increase the chance of undiagnosed cancers on CEDM. Liberman et al. reported a washout kinetic pattern presenting in 70% of infiltrating carcinomas by visual assessment of kinetic features on DCE-MRI [6]. In this study, breast cancer was significantly associated with washout kinetic pattern, especially at 2–10 min time frame. In two of the three readers’ data, CEDM showed significant association between kinetic patterns and breast pathology. One reader’s data showed no or marginal significant difference in kinetic patterns between benign and malignant breast lesions, may be due to variation in visual perception. A quantitative method for measuring breast CEDM enhancement can avoid observer-related variations in future.

CEDM costs less compared with MRI and can be used on women contraindicated for MRI scans. CEDM may be faster in image interpretation and patient examination than MRI because only two views (CC and MLO) are needed, and many conventional strengths of DM and DBT can be used during CDEM interpretation. In clinical practice, DCE-MRI of breast is advantageous because of simultaneous bilateral breast imaging and no radiation exposure. Most CEDM studies did not investigate multiple-phase imaging due to technique difficulty, excessive radiation exposure, and no commercially available software for analysis. Previous study showed the difference in contrast enhancement between MLO and CC view, which may be correlated to the kinetic change in tumor perfusion [9].

Several technical issues about this study protocol need to be addressed. In the past, patient’s movement during most DCE-MRI of breast can be a problem due to long scanning time. DCE-mammograms were obtained by direct dual-energy subtraction; hence, misregistration artifacts from motion were less problematic than in breast DCE-MRI. The mean total procedure time for stereostatic and DBT-guided breast biopsy has been reported to be 27 and 12 minutes, respectively [14]. The total examination of time of a DCE-mammogram in this study was less than 10 min, which was shorter than that of breast biopsy. Breast cancers in this DCE-mammogram study could be detected by all readers, and the diagnostic accuracy based on enhancement level was not compromised during the 8-min continuous breast compression. Moreover, the perception of a grey value can be influenced by the contrast between the lesion and its surrounding areas showing high grey values. As in DCE-MRI, the effect of background contrast enhancement in CEDM may also impact the evaluation of target lesion. Drawing region of interest (ROI) digitally would probably have been a better approach. However, this study used visual perception which was more relevant to clinical practice. The images of the affected breast are acquired only in the MLO view. A recent work of Lobbes et al. showed decline in sensitivity when a single view was acquired, so washout lesions visible earlier might not show up on subsequent single (MLO) image acquisition, resulting in issues about kinetic change of contrast agent administration [15]. An optimized protocol of CEDM with the knowledge of kinetic change can avoid diagnostic delay of breast cancer.

There are several limitations in this study. Firstly, this single-institutional prospective study involves only a small population. Secondly, the dynamic enhancing patterns in breast cancers with variable size and histopathology, showing different imaging characteristics, were not discussed [16]. Thirdly, the temporal resolution of 1–3 min, single-view MLO acquisition and five fixed time intervals for delayed contrast imaging were limited. The MLO view was chosen because it can cover more breast tissue than the CC view. The single-side kinetic analysis of a lesion is in any way justified in a clinical setting and less radiation. Fourthly, there is currently no commercial tool for quantitative analysis of signal intensity and kinetic pattern on DCE-mammogram. An iodine concentration map has shown linear relationship between the contrast-to-noise ratio and iodine concentration in phantom studies and clinical experiments [17]. Instead, this study used semi-quantitative visual interpretation, which provided a practical and easy method for clinical interpretation of CEDM.

## Conclusion

The optimal interval for peak enhancement and kinetic pattern of washout on DCE-mammogram showed promise in differentiating suspicious breast lesions. These results could be implemented as conventional bilateral acquisition protocols.

## Supporting information

S1 Table Kinetic patterns of DCE-mammogram between benign and malignant breast lesions using 2–4 and 2–10 min time intervals (reader A). (DOCX)

S2 Table Kinetic patterns of DCE-mammogram between benign and malignant breast lesions using 2–4 and 2–10 min time intervals (reader B). (DOCX)

## Abbreviations

AUC: Area under receiver operator characteristic curve
CC view: Craniocaudal view
CEDM: Contrast-enhanced digital mammogram
DCE-MRI: Dynamic contrast-enhanced magnetic resonance imaging
DCIS: Ductal carcinoma in situ
DCE-mammogram: Dynamic contrast-enhanced mammogram
DM: Digital mammogram
IDC: Invasive ductal carcinoma
MLO view: Mediolateral-oblique view
MRMC: Multi-reader multi-case
ROC: Receiver operator characteristics
