# Supplementary material for: Kinetic patterns of benign and malignant breast lesions on contrast enhanced digital mammogram
Source: PLoS One. 2020 Sep 17;15(9):e0239271. doi: 10.1371/journal.pone.0239271 (PMC7498093; doi:10.1371/journal.pone.0239271)
Supplement: S1 Table — (DOCX) [file pone.0239271.s001.docx]

**S1 Table. Kinetic patterns of DCE-mammogram between benign and malignant breast lesions using 2-4 and 2-10 min time intervals (reader A).**

| Kinetic patterns | Benign Lesions (N=75) | Malignant Lesions (N=73) | Chi-square for trend |
| --- | --- | --- | --- |
| 2-4 min |  |  | p= 0.001 |
| Persistent | 13 (17%) | 6 (8%) |  |
| Plateau | 53 (70%) | 41 (56%) |  |
| Washout | 9 (13%) | 26 (36%) |  |
| 2-10 min |  |  | p< 0.001 |
| Persistent | 11 (15%) | 4(6%) |  |
| Plateau | 44 (58%) | 28 (38%) |  |
| Washout | 20 (27%) | 41(56%) |  |
