# Supplementary material for: Kinetic patterns of benign and malignant breast lesions on contrast enhanced digital mammogram
Source: PLoS One. 2020 Sep 17;15(9):e0239271. doi: 10.1371/journal.pone.0239271 (PMC7498093; doi:10.1371/journal.pone.0239271)
Supplement: S2 Table — (DOCX) [file pone.0239271.s002.docx]

**S2 Table. Kinetic patterns of DCE-mammogram between benign and malignant breast lesions using 2-4 and 2-10 min time intervals (reader B).**

| Kinetic patterns | Benign Lesions (N=75) | Malignant Lesions (N=73) | Chi-square for trend |
| --- | --- | --- | --- |
| 2-4 min |  |  | p=0.61 |
| Persistent | 6 (8%) | 6 (8%) |  |
| Plateau | 54 (72%) | 49 (67%) |  |
| Washout | 15(20%) | 18 (25%) |  |
| 2-10 min |  |  | p=0.03 |
| Persistent | 4 (5%) | 2 (3%) |  |
| Plateau | 39 (52%) | 27 (37%) |  |
| Washout | 32 (43%) | 44 (60%) |  |
